# An Analysis of Geriatric Medicine in Malaysia-Riding the Wave of Political Change

**DOI:** 10.3390/geriatrics3040080

**Published:** 2018-11-15

**Authors:** Maw Pin Tan, Shahrul B. Kamaruzzaman, Philip Jun Hua Poi

**Affiliations:** 1 Ageing and Age-Associated Disorders Research Group, Faculty of Medicine, University of Malaya, Kuala Lumpur 50603, Malaysia; shahrulk@gmail.com (S.B.K.); philippoi@gmail.com (P.J.H.P.); 2 Division of Geriatric Medicine, Faculty of Medicine, University of Malaya, Kuala Lumpur 50603, Malaysia; 3 Sunway Medical Centre, Subang Jaya 47500, Malaysia

**Keywords:** aged, geriatrics, successful ageing, care quality, health systems, training

## Abstract

Malaysia became the centre of international attention when it democratically removed a semi-authoritarian government of 62 years during its 14th general election this year. This electoral success has provided geriatric medicine in Malaysia with a high-impact ageing icon by installing the oldest prime minister in the world. A wave of optimism for the expansion of geriatric services in Malaysia, which met with numerous challenges in the last two decades, has emerged as a result of this political change. The number of geriatrics specialists and services had begun to see slow expansions under the previous government. However, existing geriatricians will need to reassess the landscape of delivery and access to care in our rapidly growing ageing population and develop new strategies to truly expand their services. In addition to unrelenting efforts in the recruitment and training of future geriatricians, the steady expansion of the geriatric workforce should take into account the inclusion of geriatric medicine in the undergraduate training curricula of all healthcare professionals. Expansion of geriatric services will also be a cost-effective strategy to reduce the growing national healthcare budget incurred by the growing needs of an ageing population.

On 9 May 2018, Malaysia took the world by surprise by voting out its government of 62 years in its 14th general election since gaining independence from British rule in 1957. The newly elected Prime Minister, at 93, became the oldest head of state in the world. This monumental step in Malaysian and Southeast Asian politics, the birth of true democracy, has also presented a fresh opportunity to address ageism. With this major political shift, a period of re-evaluation with regard to the direction of healthcare services, particularly in the care of older persons in Malaysia, is now warranted.

Malaysia is considered an upper-middle income country. Among its population of 32 million, 2 million are aged 65 years and over, which represents 6% of its overall population [1]. While 6% would appear small compared to that of developed nations worldwide, the speed of increase in both the proportion and the absolute numbers of older persons in Malaysia is unprecedented. In the year 2010, only 5% of the Malaysian population were aged over 65 years, and by 2040, this is expected to increase to 14.5% [2]. These potentially alarming figures are fuelled by reduced birth rates, which have for the first time dipped below the replacement rate last year, as well as a rapid increase in life expectancy.

Prior to the change of government, the development of geriatric medicine in Malaysia has been challenging, with little progress made in the first decade of its existence in Malaysia [3]. However, in recent years, there has been increased investment in geriatric services, and increasing numbers of doctors specialising in geriatric medicine. Much of the expansion in geriatric services that will be seen in Malaysia in the next few years will actually have resulted from initiatives supported by the previous government. Over the past eight years, the Malaysian Ministry of Health has consistently funded at least five fellowships per year in geriatric medicine through structured subspecialty training, which leads to recognition in a National Specialist Register held by the Academy of Medicine, Malaysia. This has led to an increase in the number of practising geriatricians from 14 in 2011 to 39 in 2018. In addition to the increase in the number of geriatricians, numerous geriatric units have emerged in public hospitals throughout the country. There are now geriatric services in 11 of the 14 states, including the Federal Territory Kuala Lumpur, see Table 1. This represents a major increase from just four states in 2010.

The 39 geriatricians are distributed between public hospitals and the private sector. With one-third of the geriatricians located in the capital city of Kuala Lumpur, the geriatric services in public hospitals in all the other states comprise stand-alone units run by single-handed geriatricians in individual states, operating with a limited number of beds or in integrated general medical wards. Therefore, available geriatric services are unable to meet even a fraction of the needs of the existing older population nationwide. Hence the questions remain, can geriatric services in Malaysia expand sufficiently to cause a tangible change in the quality of care afforded to older persons and, if so, when will it eventually be possible for geriatric services to meet the needs of our older population?

When we compare the development of geriatric medicine with our closest neighbour, Singapore, one cannot help but ask, ‘what happened?’ Geriatric services in Singapore and Malaysia started at approximately the same time, but Singapore has raced ahead with the existence of well-structured training programmes for geriatricians, family medicine physicians, nurses, occupational therapists, physiotherapists, speech therapists, and social workers with the inclusion of geriatric medicine experience during undergraduate medical training [4,5]. Geriatric services in Malaysia, on the other hand, literally “got stuck for a while”, and have only started wading through mud to get to solid ground. Will the winds of political change now provide renewed energy to finally propel geriatric medicine in Malaysia forwards?

To effect positive change, the issues and challenges that held Malaysia back in developing geriatric services need to be examined and addressed. A factor in the slow expansion of the number of geriatricians in the country can be attributed to the political forces driving healthcare provision and its distribution to the more attractive and popular specialities. Geriatric medicine may appear as less “sexy” to medical graduates who have just completed their basic speciality training as many other specialities such as cardiology and gastroenterology also promise far better financial remuneration in the private sector [6]. Malaysia has also seen a rapid expansion in the number of medical colleges during the first decade of this millennium [7]. However, few of the 34 registered medical courses currently include geriatric medicine within their undergraduate curriculum. Others replace geriatrics modules with nursing home visits which may further tarnish the image of geriatric medicine [8]. Therefore, another contributing factor to the slow expansion of geriatrics is the low likelihood of medical undergraduates encountering a geriatrician, let alone being attached to a geriatric ward, throughout their medical training. Trained academic geriatricians are concentrated within one public university. While many of the other geriatricians within public hospitals do contribute as adjunct clinical lecturers to medical programmes, this is unlikely to have much impact on the 5000 medical graduates produced by the country each year [9]. The repercussions of the limitations described here need to be quantified in terms of the impact of such limited exposure to geriatric medicine among our healthcare professionals on the quality of care received by older persons in our rapidly ageing population. This is imperative, in order to inform the establishment of remedial steps to upskill the existing healthcare workforce.

Most Malaysian citizens receive healthcare through public hospitals at virtually no cost. There is a growing number of middle-class Malaysians who are now insured and seek healthcare in private hospitals, but nearly all older persons access private healthcare through out-of-pocket payments due to the unaffordable insurance premiums assigned to older clients, with others denied private insurance due to the presence of multiple high-risk conditions [10]. While community services are provided by government health clinics, these are often overcrowded, with many resorting to out-of-pocket outpatient consultations from private general practitioners. Since the 13th General Election in 2013, public healthcare has been free for all senior citizens aged 60 years and over. The rationale underlying the lack of expansion of geriatric services in both the public and private sectors do somewhat differ. Free healthcare funded fully by taxation can no longer be a sustainable funding model. This issue has been raised since the 1990s, with bills debated in Parliament to reform the health funding structure. Unfortunately, despite these efforts, the funding of the Malaysian public healthcare system has remained largely unchanged since the country’s independence [11]. The public hospitals maintain archaic hierarchical structures, which are slow to embrace change [12]. Hospital directors, who are usually senior doctors with experience in treating a large number of older persons but limited exposure to training in geriatric medicine, may find it difficult to assimilate new concepts of care amidst other competing priorities.

Many have attributed the slow pace of change in the public health system primarily to the attraction of the private sector. However, geriatric medicine has barely thrived in the private healthcare sector either, with limited financial remuneration available to private geriatricians, as consultation fees are capped by the Private Health Care Act [13]. The current payment structure in the private healthcare system severely disadvantages the geriatrician who requires long consultations with their patients. Many clinical assessments pertaining to mental status, falls assessments and frailty evaluations, have not been listed in the fees schedule, further limiting the earning potential of private geriatricians but additionally discouraging busy doctors from performing these assessments. In addition, as the health literacy of the population is limited and private hospital patients are able to access specialists directly, the lack of awareness of the role of geriatricians may limit the older adult or their family caregiver’s willingness to pay for these services out-of-pocket [14,15].

Health service provision is largely hospital-centric, with the accident and emergency department being the only portal of entry for many frail older persons. Government health clinics in urban areas are grossly oversubscribed due to the increasing burden of non-communicable diseases [16]. The sick older person is unlikely to be able to sit in the consultation room for long hours while waiting to see a doctor. This may lead to delayed presentation to medical services, while others may also choose to present to the emergency room as soon as they fall ill, acknowledging the danger of waiting. However, the older person who presents to the emergency room is at least three times more likely to be admitted to hospital [17]. With few medical schools teaching geriatric medicine, few emergency physicians have had training in caring for older adults. Therefore, negative experiences of the older client may range from inappropriate discharges to overly aggressive treatment at the end of life [18]. With the rapidly ageing population, near absence of community services, and limited expansion of public health services, it is not surprising that our hospitals, private and public, no longer have an adequate capacity to accommodate those who require hospitalization [19]. Patients who require emergency admission are regularly turned away from private and public hospitals. It is now a common occurrence for the older person to be admitted to a different hospital for each of their emergency admissions. No hospital avoidance or early discharge systems are currently in existence since hospital and community services are funded by different fiscal budgets, leading to the complete separation of care. All geriatricians are hospital-based and overwhelmed by the burden of caring for the frailest of clients who are often the victims of poor care, with little time available to address improvements to the current delivery of care.

Limited social care provisions are currently in existence, as it is expected of adult children or family members to care for their own older parents. Retirement savings are usually exhausted within three to five years of retirement, with the retirement age only just extended from 55 to 60 years five years ago [20]. The average life expectancy is now 75 years [21]. Adult children are expected to leave salaried employment to care for their dependent ageing parents if they are unable to pay for care. The average cost of 24-h care is three times the minimum wage recently announced by the new government. With the old age dependency ratio dropping rapidly, fewer families are able to meet these societal expectations. Therefore, as public hospital care remains free for senior citizens, the era of “granny dumping” has finally arrived, amidst outcries of declining moral standards. However, healthcare professionals, who are driven by performance indicators regarding the length of the hospital stay and are constantly under pressure to address delayed discharges and the lack of bed availability, may act punitively against the adult children who have “abandoned” their parents [22].

Geriatricians work in multidisciplinary teams. However, the current system supports the traditional hierarchical structure which overemphasizes the role of the doctor as the decision maker. Limited opportunities for career progression across all healthcare disciplines may lead to a less conducive working environment, where individuals feel the need to outshine their fellow colleagues rather than being mutually supportive to enhance organizational growth [23]. The hierarchical culture also prevents multidisciplinary team members from expressing their opinion or making executive decisions on individual patients, with the decision making role confined to the doctor in charge [24,25]. The hiring of healthcare professionals within the public health system still requires a vacancy to be created by the Public Services Department which is the purview of the Ministry of Human Resources. Efforts to create new salary scales for allied health professionals and nurse specialists in order to employ and retain adequately qualified nurse specialists, occupational therapists, speech therapists, and psychologists to name but a few, have met with limited success [26]. Therefore, the geriatrician struggles to assemble multidisciplinary teams and retain multidisciplinary team members, let alone operate as multidisciplinary teams which know how to work as teams.

Future service development now needs to plan for a service that not only effectively influences the care of older adults but also grows at a pace faster than the relentless increase in older patients. Development needs to take into account human resource, culture change, structural changes, and service delivery. All the above is indeed challenging, but there is no alternative. Without addressing the increasing healthcare needs associated with population ageing, our country may not be able to break out of the middle-income trap that has held it back from attaining the much-coveted higher income status over the past two decades. The demographic dividend that our country should have prospered from has more or less ended [27].

There is great potential for expansion of the geriatrics workforce with strong commitment to recruit and train future geriatricians. The number of individuals entering subspecialty training in geriatric medicine should be determined solely by three factors: the number of individuals with the right aptitude and desire, the number of geriatricians, and the number of training centres. The requirement for the award of fellowships for training should be reviewed, as subspecialty training can occur as long as doctors in training, who hold permanent posts and receive salaries from the ministry of health regardless, are appropriately placed in posts attached to geriatric services. These posts would otherwise be occupied by individuals who are not necessarily interested in pursuing geriatric medicine as a career, placed in these posts by administrators within the Ministry of Health. Those interested in geriatric medicine should be incentivised through a fast-track mechanism where any administrative and political delay in entering training is minimized. With the massive increase in the numbers of medical graduates, our country is no longer short of doctors [28]. The more explicit entry criteria offered by the National Postgraduate Curriculum for Internal Medicine will help ensure trainees should no longer experience a delay in their entry into subspecialty training; provided the unequal distribution of human resources between subspecialties is addressed. Subspecialty geriatric training in Malaysia currently requires three years. Only geriatricians working in the public sector are trainers, and these comprise 60% of the total geriatrician population. Therefore, a simple projection assuming all public sector geriatricians train one geriatrician every three years will see to the achievement of the Canadian 2011 levels of 0.5 per 10,000 population aged 65 years or over by 2026 and UK targets of 0.85 per 10,000 population aged 65 years or over by 2030, see Figure 1 [29,30]. Human resource expansion also needs to include other professionals allied to geriatric medicine, which include nurses, physiotherapists, occupational therapists, speech therapists, dietitians, psychologists, and social workers. The Public Services Department should consider revising the availability of posts for allied health professionals to enable more equitable systems that are both sensitive and responsive to local needs.

Exposure to geriatric medicine needs to be mandated for undergraduate courses for all healthcare professionals [31]. However, it may not be possible to ensure sufficient exposure to geriatric services initially. Therefore, the minimum requirement should be for all undergraduate courses for healthcare professionals to include a geriatrics curriculum [32]. As all such courses require accreditation by the Malaysian Qualifications Agency, this can almost be implemented overnight by mandating the inclusion of a geriatrics curriculum in all undergraduate courses for healthcare professionals as a criterion for accreditation [33,34]. These courses should then be incentivized through ratings and funding availability to include clinical geriatrics exposure, which will eventually be made compulsory once clinical services have expanded adequately. Early exposure to geriatric medicine will ensure that future healthcare professionals will graduate with the requisite skills to care for older patients, reducing the level of potential harm to patients experienced by all older persons seeking medical attention today [35]. In addition, more medical graduates and allied health professionals are likely to take up geriatric medicine as a subspecialty or specialist interest if they have had the opportunity to meet and be inspired by those caring for older persons early on in their careers [36]. As a positive step toward the right direction, the National Postgraduate Curriculum for Internal Medicine is due to be launched in 2019 with the inclusion of a geriatrics component.

The hierarchical working culture which continues to plague the healthcare system lends poorly to the development of geriatric medicine and effective teamwork. Therefore, the re-examination and establishment of organizational structures which reward meritocracy rather than seniority are imperative. Training regarding effective leadership and teamwork needs to be included in undergraduate, postgraduate, and continuing professional development courses and effective disciplinary procedures need to be put in place to ensure such a culture is no longer perpetuated [37]. Instead, effective teams are acknowledged and rewarded. Geriatric medicine will only thrive if the multidisciplinary team adopts a flat hierarchy where all members of the team are empowered to contribute information to aid decision making [38]. Colleagues from more established subspecialties must be willing put aside the “they must fight for it and wait for their turn like I did” attitude and to consider the interest and needs of the patient first and foremost.

In this climate of change in our nation, policymakers and hospital management need to acknowledge that geriatric services pay for themselves through cost-effectiveness. The comprehensive geriatrics assessment (CGA) is associated with reduced mortality at a median follow-up of 12 months, reduced likelihood of institutional care, and improved cognition. The key components of a CGA include coordinated multidisciplinary assessment, specialist geriatric medicine review, identification of medical, physical, social and psychological problems, and formation of a care and rehabilitation plan [39]. The current approach of equating geriatrics to general medicine or only allowing the practice of geriatric medicine through consults is counterproductive. Available evidence indicates that care provided by roving teams has poorer outcomes, with the evidence supporting coordinated multidisciplinary teams delivered through dedicated specialist beds [40]. In addition, the development of new models of care needs to be considered, for instance, the use of advanced practice nurses who collaborate with geriatricians to more widely disseminate geriatric care to the population. In the current climate, where the new government is crying foul of excessive debts which will lead to continued economic hardship for the next few years, rapid expansion in geriatric services is likely to help “balance the books” with regards to healthcare funding.

Addressing the imbalance in funding between health and social care whereby healthcare is free for senior citizens, fully funded by taxation, while social care remains almost entirely out-of-pocket with the expectation that adult children and family members meet the cost, is likely to represent the biggest challenge in health and long-term care reforms [41,42]. While universal insurance coverage for both health and long-term care funded by taxation would seem the obvious solution, its implementation is likely to be unpopular and may be impeded by objections from numerous sources [11]. Furthermore, lessons from the UK National Health Service suggest that a universal national insurance system is unlikely to be sustainable [43]. No clear solution currently exists to address the matter of health and long-term care funding for older adults [44]. In this regard, we are perhaps starting on a level pegging with other developed economies which are in agreement that the approach of “providing for the older persons’ needs” is not a sustainable one [45]. However, the effective solution for this politically sensitive area remains elusive. Armed with the knowledge of “how not to do it” by learning from other “failed” systems, what we have is a clean slate from which we can build a system that does not repeat anybody else’s mistakes [46].

Having a 93-year-old Prime Minister provides the nation with a good model of successful ageing, and may well be the effective driving force for wholesale attitudinal change and the implementation of policies for older persons which are in existence. The road ahead in making up for lost time and opportunities to reform health services towards adequately meeting the needs of the ageing population remains daunting. Issues associated with lack of popularity, limited early exposure of the workforce, hierarchical organisational structures, and the absence of social care funding will need definitive action, in order to enable accelerated expansion of geriatric medicine as a subspecialty.

## Figures and Tables

**Figure 1:**
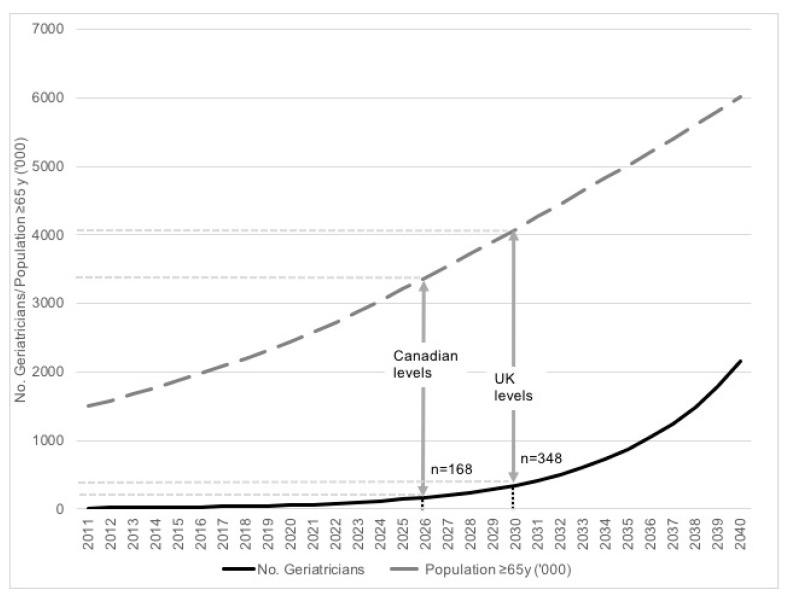
Projection of Older Population and Number of Geriatricians in Malaysia from 2011–2040. The population projections for the total number of Malaysians aged 65 years and over from 2011 to 2040 (broken grey line) with a simulated model of expected number of geriatricians in Malaysia if 60% of geriatricians trained one new geriatrician every three years (solid black line). The vertical grey arrows indicate the timepoints where the number of geriatricians will meet Canadian levels, and United Kingdom targets levels.

**Table 1:** Distribution of geriatricians according to the states of Malaysia.

State	Number of Geriatricians *	Population Aged ≥ 65 Years (‘000) **
West Malaysia		
Northern States		
Perlis	None	21.6
Kedah	1 Public	158.7
Penang	1 Public, 2 Private	141.3
Perak	2 Public, 2 Private	246.5
Central States		
Federal Territory	11 Public, 3 Private, 1 Retired	120.2
Selangor	4 Public, 2 Private	306.1
Negeri Sembilan	1 Public	81.6
Southern States		
Johor	1 Public, 1 Private	248.3
Melaka	1 Public, 1 Private	71.1
East Coast		
Pahang	1 Public (visiting geriatrician from Selangor)	110.8
Kelantan	None	113.8
Terengganu	None	65.4
East Malaysia		
Sarawak	1 Public, 1 Private	192.7
Sabah	1 Public, 1 Private	123.4

* indicates total number of geriatricians working in the public and private sectors as of September 2018; ** Department of Statistics Malaysia Population Estimates [2].

## References

[1] DOSM (2018). Current Population Estimates.

[2] DOSM (2016). Population Projections (Revised).

[3] Poi P.J., Forsyth D.R., Chan D.K. (2004). Services for older people in Malaysia: Issues and challenges. Age Ageing.

[4] Koh G.C.H. (2007). A review of geriatric education in Singapore. Ann. Acad. Med. Singap..

[5] Chua M.P.W., Tan C.H., Merchant R., Soiza R.L. (2008). Attitudes of first-year medical students in Singapore towards older people and willingness to consider a career in geriatric medicine. Ann. Acad. Med. Singap..

[6] Robbins T.D., Crocker-Buque T., Forrester-Paton C., Cantlay A., Gladman J.R.F., Gordon A.L. (2011). Geriatrics is rewarding but lacks earning potential and prestige: Responses from the national medical student survey of attitudes to and perceptions of geriatric medicine. Age Ageing.

[7] Lim V.K.E. (2008). Medical education in Malaysia. Med. Teach..

[8] Secretariat M.M.C. (2016). The Malaysian Medical Council Annual Report 2015.

[9] Secretariat M.M.C. (2018). Second Schedule, Medical Act 1971.

[10] NoorAni A., Rajini S., Balkish M.N., Noraida M.K., Smaria A., Fadhli M.Y., Jabrullah A.H., Tahir A. (2018). Morbidity patterns and healthcare utilisation among older people in Malaysia: 1996–2015. Public Health.

[11] Chua H.T., Cheah J.C.H. (2012). Financing Universal Coverage in Malaysia: A case study. BMC Public Health.

[12] Thomas S., Beh L., Nordin R. (2011). Bin Health care delivery in Malaysia: Changes, challenges and champions. J. Public Health Africa.

[13] (2013). Private Healthcare Facilities and Services (Private Hospitals and Other Private Healthcare Facilities) (Amendment) Order 2013.

[14] Yunus N.M., Manaf N.H.A., Omar A. (2017). Determinants of healthcare utilisation among the elderly in Malaysia. Inst. Econ..

[15] Dawood O.T., Hassali M.A., Saleem F., Ibrahim I.R., Abdulameer A.H., Jasim H.H. (2017). Assessment of health seeking behaviour and self-medication among general public in the state of Penang, Malaysia. Pharm. Pract..

[16] Saidi S., Milnes L.J., Griffiths J. (2018). Fatalism, Faith and Fear: A case study of self-care practice among adults with type 2 diabetes in urban Malaysia. J. Clin. Nurs..

[17] Mohd Mokhtar M.A., Pin T.M., Zakaria M.I., Hairi N.N., Kamaruzzaman S.B., Vyrn C.A., Hua P.P.J. (2015). Utilization of the emergency department by older residents in Kuala Lumpur, Malaysia. Geriatr. Gerontol. Int..

[18] Banerjee J., Conroy S., Cooke M.W. (2013). Quality care for older people with urgent and emergency care needs in UK emergency departments. Emerg. Med. J..

[19] Safurah J., Kamaliah M.N., Khairiyah A.M., Nour Hanah O., Healy J., Kalsom M., Zakiah M.S. (2012). Malaysia Health System Review.

[20] Samad S.A., Mansor N. (2017). Population Ageing and Social Protection in Malaysia. Malays. J. Econ. Stud..

[21] DOSM Abridged Life Tables, Malaysia, 2015–2017. https://www.dosm.gov.my/v1/index.php?r=column/cthemeByCat&cat=116&bul_id=dkdvKzZ0K1NiemEwNlJteDBSUGorQT09&menu_id=L0pheU43NWJwRWVSZklWdzQ4TlhUUT09.

[22] Sooryanarayana R., Choo W.Y., Hairi N.N., Chinna K., Bulgiba A. (2015). Insight into elder abuse among urban poor of Kuala Lumpur, Malaysia—A middle-income developing country. J. Am. Geriatr. Soc..

[23] Samsudin E.Z., Isahak M., Rampal S. (2018). The prevalence, risk factors and outcomes of workplace bullying among junior doctors: A systematic review. Eur. J. Work Organ. Psychol..

[24] Boev C., Xia Y. (2015). Nurse-Physician Collaboration and Hospital-Acquired Infections in Critical Care. Crit. Care Nurse.

[25] Crowe S., Clarke N., Brugha R. (2017). ‘You do not cross them’: Hierarchy and emotion in doctors’ narratives of power relations in specialist training. Soc. Sci. Med..

[26] Hameed L.M., Nor F.M. Public and Private Shares in the Distribution of Doctors in Malaysia. Proceedings of the Conference on Management and Muamalah.

[27] Ismail N.W., Rahman H., Hamid T. (2015). Does population aging affect economic growth in Malaysia. Pros. Perkem.

[28] Wong R.S.Y., Abdul Kadir S.Y. (2017). Medical education in Malaysia: Quality versus quantity. Perspect. Med. Educ..

[29] Hogan D.B., Borrie M., Basran J.F.S., Chung A.M., Jarrett P.G., Morais J.A., Peters E., Rockwood K.J., St. John P.D., Sclater A.L. (2012). Specialist Physicians in Geriatrics—Report of the Canadian Geriatrics Society Physician Resource Work Group. Can. Geriatr. J..

[30] Fisher J.M., Garside M., Hunt K., Lo N. (2014). Geriatric medicine workforce planning: A giant geriatric problem or has the tide turned?. Clin. Med..

[31] Bardach S.H., Rowles G.D. (2012). Geriatric education in the health professions: Are we making progress?. Gerontologist.

[32] Masud T., Blundell A., Gordon A.L., Mulpeter K., Roller R., Singler K., Goeldlin A., Stuck A. (2014). European undergraduate curriculum in geriatric medicine developed using an international modified Delphi technique. Age Ageing.

[33] Bhardwaj A., Kavitha N., Ibrahim S. (2017). Malaysian Medical License Examination (MMLE): Is This A Way Forward?. Educ. Med. J..

[34] Burch V.C., Adnan N.A.M., Afolabi B.B., Ismail Z., Jafri W., Olapede-Olaopa E.O., Otieno-nyunya B., Supe A., Togoo A., Vargas A. (2006). Accreditation of undergraduate medical training programs: Practices in nine developing countries as compared with the United States. Educ. Health.

[35] Sourdet S., Lafont C., Rolland Y., Nourhashemi F., Andrieu S., Vellas B. (2015). Preventable iatrogenic disability in elderly patients during hospitalization. J. Am. Med. Dir. Assoc..

[36] Verma P., Ford J.A., Stuart A., Howe A., Everington S., Steel N. (2016). A systematic review of strategies to recruit and retain primary care doctors. BMC Health Serv. Res..

[37] Tran V. (2015). Dealing with bullying and harassment: A practical guide for Australasian emergency medicine trainees. Emerg. Med. Australas.

[38] Weller J., Boyd M., Cumin D. (2014). Teams, tribes and patient safety: Overcoming barriers to effective teamwork in healthcare. Postgrad. Med. J..

[39] Ellis G., Whitehead M.A., O’Neill D., Langhorne P., Robinson D. (2014). Comprehensive geriatric assessment for older adults admitted to hospital. Cochrane Database Syst. Rev..

[40] Stott D.J., Quinn T.J. (2017). Principles of rehabilitation of older people. Medicine.

[41] Wei K.C., Lee C., Mahendran R., Lim C.G. (2012). Improving mental health care for people with an intellectual disability in Singapore: Bridging the health-social care divide. Singap. Med. J..

[42] Zhu H., Walker A. (2018). The gap in social care provision for older people in China. Asian Soc. Work Policy Rev..

[43] Alderwick H., Ham C. (2017). Sustainability and transformation plans for the NHS in England: What do they say and what happens next?. BMJ.

[44] Goodwin N., Dixon A., Anderson G., Wodchis W. (2014). Providing Integrated Care for Older People with Complex Needs: Lessons from Seven International Case Studies.

[45] Alley D.E., Asomugha C.N., Conway P.H., Sanghavi D.M. (2016). Accountable health communities—Addressing social needs through Medicare and Medicaid. N. Engl. J. Med..

[46] Thornicroft G., Alem A., Santos R.A., Barley E., Drake R.E., Gregorio G., Hanlon C., Ito H., Latimer E., Law A. (2010). WPA guidance on steps, obstacles and mistakes to avoid in the implementation of community mental health care. World Psychiatry.

